# Exposure to DMSO during infancy alters neurochemistry, social interactions, and brain morphology in long‐evans rats

**DOI:** 10.1002/brb3.2146

**Published:** 2021-04-10

**Authors:** Zachary Rabow, Taryn Morningstar, Megan Showalter, Hailey Heil, Krista Thongphanh, Sili Fan, Joanne Chan, Verónica Martínez‐Cerdeño, Robert Berman, David Zagzag, Evgeny Nudler, Oliver Fiehn, Mirna Lechpammer

**Affiliations:** ^1^ Department of Pathology and Laboratory Medicine School of Medicine University of California Davis Sacramento CA USA; ^2^ NIH West Coast Metabolomics Center University of California Davis Davis CA USA; ^3^ MIND Institute University of California Davis Sacramento CA USA; ^4^ Institute for Pediatric Regenerative Medicine and Shriners Hospital for Children of Northern California Sacramento CA USA; ^5^ Department of Neurological Surgery University of California Davis Sacramento CA USA; ^6^ Departments of Pathology and Neurosurgery Division of Neuropathology NYU Langone Medical Center New York NY USA; ^7^ Howard Hughes Medical Institute New York University School of Medicine New York NY USA; ^8^ Department of Biochemistry & Molecular Pharmacology New York University School of Medicine New York NY USA; ^9^ Pathology Foundation Medicine, Inc. Cambridge MA USA

**Keywords:** glial cells, metabolomics, neurochemistry, neuropharmacology

## Abstract

**Introduction:**

Dimethyl sulfoxide (DMSO) is a widely used solvent to dissolve hydrophobic substances for clinical uses and experimental in vivo purposes. While usually regarded safe, our prior studies suggest changes to behavior following DMSO exposure. We therefore evaluated the effects of a five‐day, short‐term exposure to DMSO on postnatal infant rats (P6‐10).

**Methods:**

DMSO was intraperitoneally injected for five days at 0.2, 2.0, and 4.0 ml/kg body mass. One cohort of animals was sacrificed 24 hr after DMSO exposure to analyze the neurometabolic changes in four brain regions (cortex, hippocampus, basal ganglia, and cerebellum) by hydrophilic interaction liquid chromatography. A second cohort of animals was used to analyze chronic alterations to behavior and pathological changes to glia and neuronal cells later in life (P21‐P40).

**Results:**

164 metabolites, including key regulatory molecules (retinoic acid, orotic acid, adrenic acid, and hypotaurine), were found significantly altered by DMSO exposure in at least one of the brain regions at P11 (*p* < .05). Behavioral tests showed significant hypoactive behavior and decreased social habits to the 2.0 and 4.0 ml DMSO/kg groups (*p* < .01). Significant increases in number of microglia and astrocytes at P40 were observed in the 4.0 ml DMSO/kg group (at *p* < .015.)

**Conclusions:**

Despite short‐term exposure at low, putatively nontoxic concentrations, DMSO led to changes in behavior and social preferences, chronic alterations in glial cells, and changes in essential regulatory brain metabolites. The chronic neurological effects of DMSO exposure reported here raise concerns about its neurotoxicity and consequent safety in human medical applications and clinical trials.

## Graphical abstract

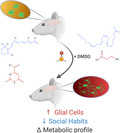

## 1 INTRODUCTION

Dimethyl sulfoxide (DMSO) is regarded as a safe solvent, commonly used in in vivo experiments. It is also widely available as an over‐the‐counter, topical pain‐relieving agent, and can be purchased in large quantities with no regulation. Additionally, DMSO is a cryopreservative that is re‐infused along with stem cells during autologous bone marrow transplantations (Hanslick et al., 2009). DMSO rapidly penetrates the skin and can transport drugs and other molecules that would otherwise not cross the skin. When applied to the skin, DMSO has local anesthetic properties and reduces swelling (Maryland, 1967). Experimentally, DMSO has been used to treat intestinal, renal, and cerebral ischemia, and has been shown to suppress central nervous system (CNS) injury by scavenging inflammation‐triggering free radicals (De la Torre et al., 1975; Kedar et al., 1983; Kharasch & Thyagarajan,; Little et al., 1983; Ravid et al., 1983). DMSO is fully miscible with other aqueous substances and can form chemical associations with many different molecules and matrixes, including metal ions, drugs, and components of tissue, blood, plasma, and spinal fluid (Wong & Reinertson, 1984).

Dimethyl sulfoxide crosses the blood–brain barrier and has been effective in the treatment of traumatic brain edema by reducing the increase in intracranial pressure and by elevating cerebral blood flow without altering blood pressure (Brown et al., 1980; Camp et al., 1981; De la Torre et al., 1975; Ikeda & Long, 1990; Karaca et al., 1991). Dimethyl sulfoxide is metabolized to dimethyl sulfide (DMS) and dimethyl sulfone (DMSO_2_). Unmetabolized DMSO is the most abundant form found in the body after exposure. DMSO is found in tissue, blood, feces, and urine regardless of application route (Wong & Reinertson, 1984).

The United States Federal Drug Administration (FDA) approved the use of DMSO for the treatment of interstitial cystitis under the trade name "Rimso‐50" (Willhite & Katz, 1984). The toxicology studies, which demonstrated the perceived safety of DMSO, did not look at long‐term effects from brief exposure to the compound. The high doses of DMSO that are tolerated by various application routes have led to DMSO being deemed relatively nontoxic and safe (Table 1) (Leake, 1966). More advanced technology has been developed to examine cellular, epigenetic, and metabolic changes since the 1970s when DMSO was approved by the FDA, so the safety of DMSO in both the clinical setting and its use in research should be revisited (Crawley, 2004; Yuan et al., 2016).

**TABLE 1:** LD50 values in g/kg body weight for DMSO (Amended from Leake, 1966)

Species	Dermal	*per os*	*i.v*.	*s.c*.
Application route				
Mouse	50	21–28	4–9	15–25
Rat	40	15–28	5–8	13
Monkey	>11	>10	2–3	—

Note

LD50 values for various species with different application or exposure routes. per os—oral administration; i.v.—intravenous injection; s.c.—subcutaneous injection.

Autologous stem cell transplants require the use of cryopreservatives to protect cells from freezing damage. DMSO is the standard and most commonly used cryoprotective agent (Al‐Anazi, 2012). Hanslik et al. (2009) found that the average dose of DMSO was 0.63 ml/kg when used as a cryoprotectant for medical purposes. When DMSO is transfused into patients, it has several well‐identified side effects, including nausea, vomiting, abdominal cramps, and headaches. Acute neurological abnormalities immediately following the infusion of stem cells suspended in the cryopreservative DMSO have been reported in some patients (Chen‐Plotkin et al., 2007; Davis et al., 1990; Hanslick et al., 2009; Martín‐Henao et al., 2010; Santos et al., 2003).

DMSO is used in the process of in vitro fertilization for embryo cryopreservation, as well as the treatment of palmar–plantar erythrodysesthesia syndrome (hand–foot syndrome), which is a common side effect in cancer treatments (Lechpammer et al., 2016). To our knowledge, no papers have been published to examine the behavioral and cognitive effects of neonatal DMSO exposure in rodents. Two published studies have reported behavioral effects in rodents following DMSO exposure; however, these studies were performed in adult rats, and it remained unclear whether DMSO has caused permanent changes in animals (Authier et al., 2002; Fossom et al., 1985). Since fetuses and neonates are exposed to DMSO in the practice of pediatric medicine or during treatment of the mother and through her use of topical products that contain DMSO, we see this practice as a potential area of unrecognized neurotoxicological exposure that might add to psychiatric complications in childhood such as decreased social behavior that could present similar to Asperger's disorder (Verheijen et al., 2019).

The purpose of this study was to assess the effects of DMSO on neonatal male rats following exposures to clinically relevant doses. DMSO exposure was performed during the period of brain development vulnerability in LE neonatal rats, which corresponds to the critical myelination time periods in humans lasting from the last trimester through early childhood (Downes & Mullins, 2014; Semple et al., 2013). To that end, we evaluated the effects of DMSO on metabolome of the brains after short‐term neonatal exposure from postnatal days 6–10 (P6‐10). Additionally, we assessed chronic changes to behavior, social preferences, and brain morphology in adult rats after neonatal exposure. The limitations of this study are only male rats were used, and longitudinal sampling was not able to be performed to investigate metabolic changes over time. Here, we present the first investigation of DMSO during developmental time periods in rats with follow‐up studies later in life.

## 2 RESULTS AND DISCUSSION

### 2.1 Chronic changes to social behavior later in life after neonatal exposure to DMSO

**FIGURE 1:**
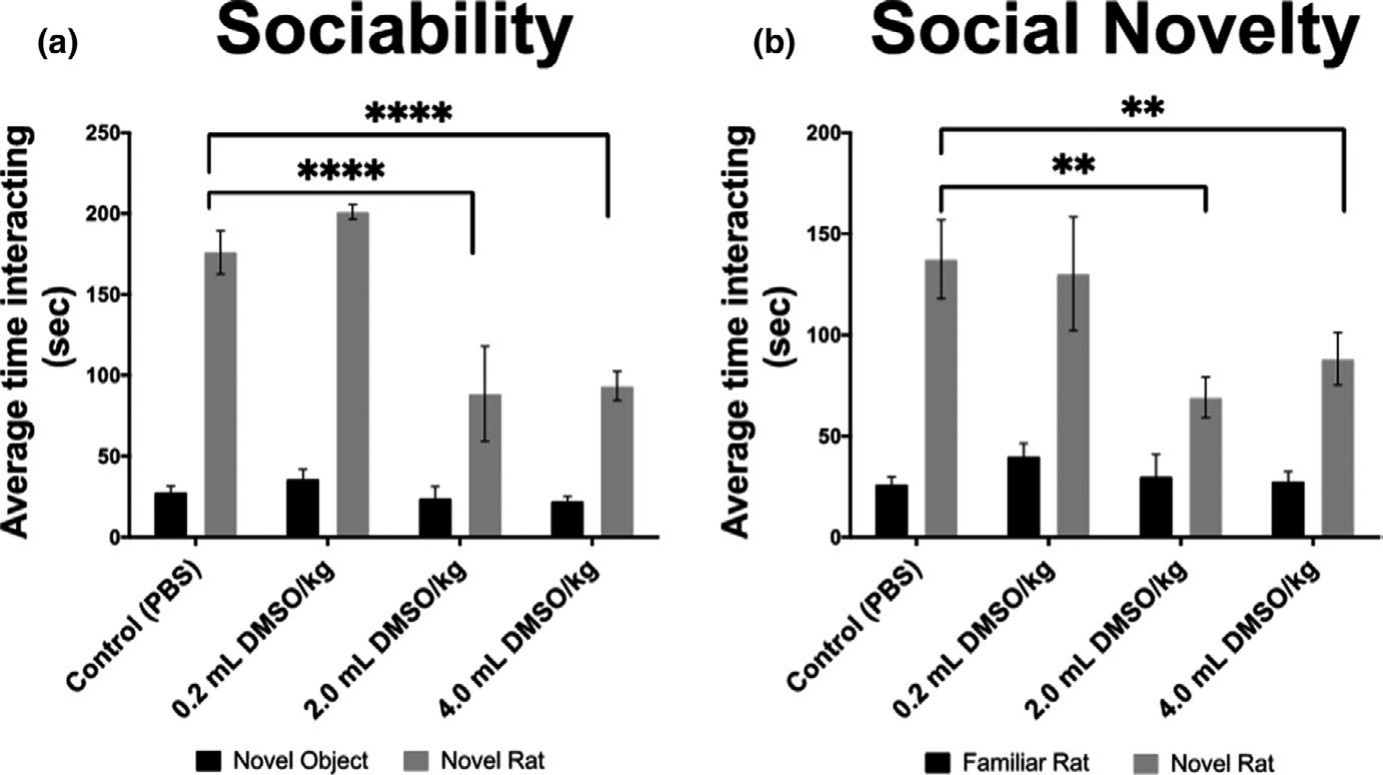
Behavior assessment for adult rats following neonatal exposure to DMSO (*n* = 30). Experimental groups were prepared and submitted to behavioral assessments as described in the methods. Depicted are behavioral patterns for sociability and social novelty assessed at P32. Significant decreases in interaction time were observed following DMSO exposure in both the sociability and social novelty test. Values are represented as means ± *SEM*.; sociability tests for both the 2.0 and 4.0 ml DMSO/kg groups, *p* < .0001. Social novelty tests for the 2.0 ml DMSO/kg group, *p* = .0041, and the 4.0 ml DMSO/kg group, *p* = .0085. **p < .01 versus sham‐treated control (PBS); ****p < .0001 versus sham‐treated control (PBS)

We evaluated the effects of a five‐day, short‐term exposure of DMSO during the critical phase of myelination in neonatal Long‐Evans (LE) rats (P6‐10). DMSO was intraperitoneally (IP) injected twice per day for five days at 0.2, 2.0, and 4.0 ml/kg body mass (Downes & Mullins, 2014). Behavior was assessed at P21 using the open‐field locomotion test. Social preferences were assessed at P32 using the three‐chambered social approach test. No changes were observed in locomotion behavior as assessed by the open‐field test (Figure S1); however, social preferences were altered in the 2.0 and 4.0 ml DMSO/kg groups (*p* < .0001) (Figure 1a). Both groups showed a significant decrease in sociability as indicated by less time spent with a social stimulus (novel rat) than a nonsocial object (empty wire cage). Additionally, in the preference for social novelty, the preference for interacting with a novel rat over a familiar rat was significantly reduced in both the 2.0 ml DMSO/kg group (*p* = .004) and the 4.0 ml DMSO/kg group (*p* = .009) compared with the sham‐treated controls (Figure 1b).

These results suggest prolonged effects on behavior and social cognition following brief exposure to DMSO early in life.

### 2.2 Astrocytes and microglia increase in a dose‐dependent manner following exposure to DMSO

**FIGURE 2:**
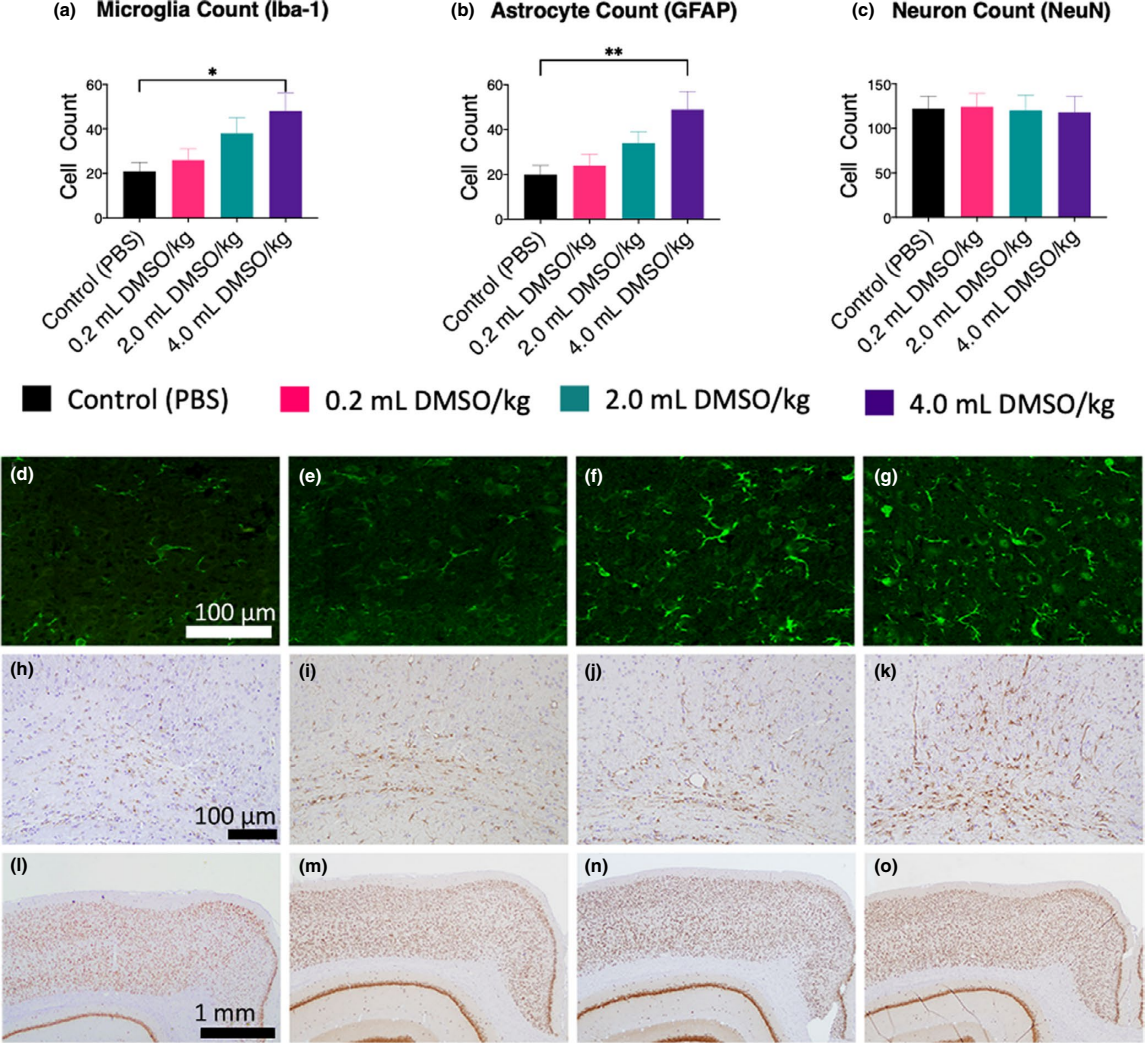
Immunohistochemical analysis of microglia, astrocytes, and cortical neurons in adult rats (P40) following neonatal exposure to DMSO at P6‐10 (*n* = 24, 6/group). Tissue was collected and prepared as described in the methods. Data were collected in a randomized, blinded manner by two neuroscientists. (a–c) Quantitative analysis of microglia, astrocytes, and neurons. (d–g) Representative immunohistochemical stains showing an increase in microglia (Iba‐1 stain) with increasing DMSO doses; (d) control; (e) 0.2 ml DMSO/kg; (f) 2.0 ml DMSO/kg; (g) 4.0 ml DMSO/kg (size bar = 100 µm). (h–k) Representative immunohistochemical stains showing an increase in astrocytes (GFAP stain) with increasing DMSO doses; (h) control; (i) 0.2 ml DMSO/kg; (j) 2.0 ml DMSO/kg K); 4.0 ml DMSO/kg (size bar = 100 µm). (l–o) Representative immunohistochemical stains showing no change in cortical neurons (NeuN stain) with exposure to DMSO; (l) control; (m) 0.2 ml DMSO/kg; (n) 2.0 ml DMSO/kg; (o) 4.0 ml DMSO/kg (size bar = 1 mm). Values are represented as means ± *SEM*; *p < .05 versus sham‐treated control (PBS); **p < .01 versus sham‐treated control (PBS)

Brain morphology was assessed at P40, 30 days after DMSO exposure was stopped. No gross morphological brain changes were observed. Microglia, astrocytes, and cortical neurons were assessed (*n* = 24, 6 per group). Immunohistochemical analysis of microglia showed a DMSO dose‐dependent increase in the number of microglia. Significant increases in Iba‐1‐expressing microglia were observed in the 4.0 ml DMSO/kg group (*p* = .019) (Figure 2a). Astrocytes (assessed by GFAP stain) had a similar response, and significant increases were observed in the 4.0 ml DMSO/kg group (*p* = .0065) (Figure 2b). No changes were seen in number of cortical neurons (Figure 2c).

Increase in the number of microglia and astrocytes is common following neurological injury. Microglia play a fundamental role in phagocytosis and debris clearance in the CNS via phagocytic clearance by facilitating the reorganization of neuronal circuits and initiating repair mechanisms (Neumann et al., 2009). Chronic assaults to the CNS lead to microglia developing a hypertrophic morphology instead of a retracted or amoeboid morphology. Various mental and behavioral issues are linked to the elevation of hypertrophic microglia (Calcia et al., 2016). The increase in microglia observed after DMSO exposure provides a possible explanation for the alterations in normal behavior and social preferences. Following CNS injury, astrocytes produce cytokines, chemokines, and anti‐inflammatory mediators to respond to injured or dying cells (Burda et al., 2016; Liauw et al., 2008). In parallel, large areas of active neural tissue undergo astrogliosis due to the destruction of nearby neurons. The activation of astrocytes following injury leads to increased survival of neurons by extending hypertrophic processes to the traumatized neurons (Bylicky et al., 2018). This cell–cell interaction leads to an increase in glycoproteins and transcriptional activators that lead to axonal sprouting (Campbell et al., 1985; Carmichael et al., 2017). Damaged and destroyed neurons may explain the hypoactive and irregular social preferences and behavior observed in our study.

### 2.3 DMSO accumulates in various brain regions

**FIGURE 3:**
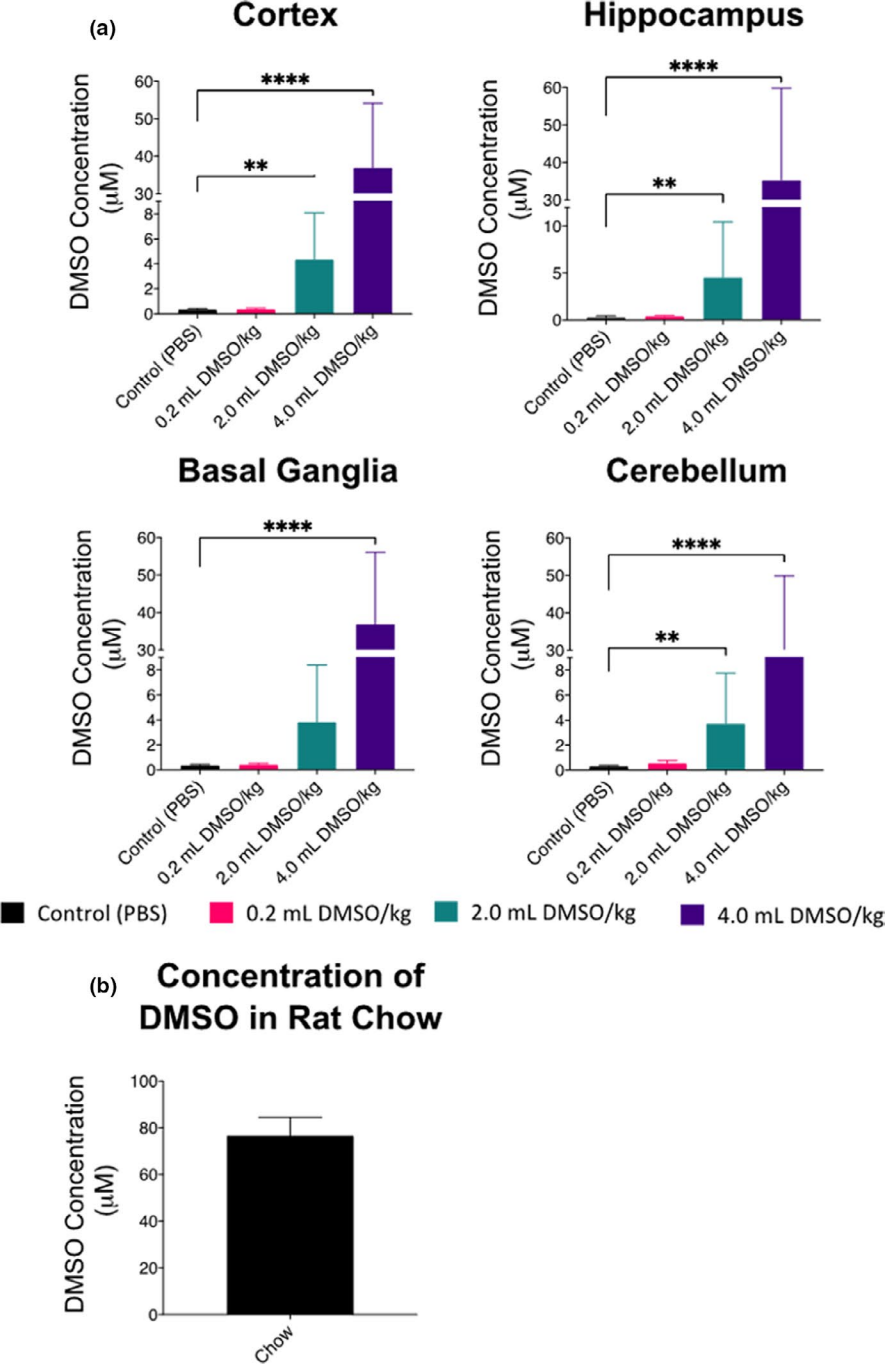
Quantification of DMSO by HILIC‐Q Exactive HF MS/MS in the brain 24 hr after exposure (*n* = 20, 5 per group). (a) Concentration of DMSO by region and dose. (b) Concentration of DMSO in standard chow; values are represented as means ± *SEM*; **p < .01 versus control (PBS); ****p < .0001 versus control (PBS)

Animals (*n* = five rats per treatment group, 20 total) were sacrificed for brain metabolome analysis at postnatal day 11 (P11), 24 hr after the administration of DMSO stopped. The presence and abundance of metabolites were assessed by hydrophilic interaction chromatography combined with high‐resolution mass spectrometry (HILIC‐Q Exactive HF MS/MS, see Method Section). We first investigated the data set for the detection of the target molecule DMSO. DMSO was detected in tissue from all four analyzed brain regions: cortex, hippocampus, basal ganglia, and cerebellum. We found increasing, dose‐dependent mean levels of DMSO in the different brain regions ranging from 0.3 to 0.5 µM DMSO in the 0.2 ml DMSO/kg group to 3–4 µM DMSO in the 2.0 ml DMSO/kg group and finally 27–37 µM DMSO in the 4.0 ml DMSO/kg group. DMSO was significantly higher in the 4.0 ml/kg groups of all four analyzed brain regions (*p* < .0001) and significantly higher in the 2.0 ml/kg group in the cortex, hippocampus, and cerebellum (*p* < .001) compared with the controls. Interestingly, even the sham‐treated control group showed low levels of DMSO present as well, with a mean concentration of 0.3 µM DMSO for all regions (Figure 3a). We investigated the origin of DMSO in untreated rats and found that the rat chow itself contained 76 µM DMSO (Figure 3b). Interestingly, as these were neonatal animals and still nursing, only the dam was ingesting the chow, and thus, the DMSO was transmitted via dam's milk.

Through IP administration, DMSO is absorbed into the mesenteric blood supply and then carried to the liver and subjected to hepatic metabolism. The amount of DMSO that was passed from the dam to the pups via milk is unknown. DMSO levels detected in the brains of the control rats were likely transported through small intestinal absorption from chow in the dam, and then metabolized in the liver before passing to the bloodstream, and, ultimately, the dam's milk. The DMSO detected in the control pups likely derived from gastrointestinal absorption of the dam's milk, which was introduced due to maternal consumption of the chow (Turner et al., 2011).

Our results reported here expand on the prior work that investigated DMSO levels in plasma after exposure. Yellowlees et al. (1980) found that an oral dose of 1 g DMSO/kg body weight (0.91 ml DMSO/kg) resulted in peak plasma concentrations in 4 to 6 hr, with detectable levels present for 400 hr (Yellowlees et al., 1980). Our findings support reports by Hanslick et al. (2009) who observed differences in relative degeneration severity of different brain regions, suggesting that cell‐specific transporters may play a crucial role in responding to the damage induced by DMSO (Hanslick et al., 2009).

We found that the 0.2 ml DMSO/kg group had nearly the same levels of DMSO incorporated as the sham‐treated animals. As our instruments were sensitive enough to detect even minute quantities of DMSO (Figure S2), we hypothesize that there might be an inflection point of the glymphatic and circulatory system that clears low amounts of DMSO from the brain as the relationship between administered DMSO and DMSO found in regions of the brain seems characteristic of a saturation curve, where uptake and clearance are not proportional.

### 2.4 The brain metabolome is drastically altered by low doses of DMSO

Four regions of the rats’ brain (cortex, hippocampus, basal ganglia, and cerebellum) were macrodissected and analyzed at P11 (*n* = 20, 5 per group) for metabolic alterations following five days of DMSO exposure. These regions were chosen for their well‐defined functions related to behavior, social interactions, and motor functions. Metabolic alterations were assessed by using hydrophilic interaction liquid chromatography coupled to orbital ion trap MS/MS (HILIC‐Q Exactive HF MS/MS). 483 structurally identified metabolites were detected by matching retention times, accurate mass, and MS/MS spectra to authentic compounds and entries in the MoNA and NIST17 databases. Data have been uploaded to the NIH MetabolomicsWorkbench.org database.

**FIGURE 4:**
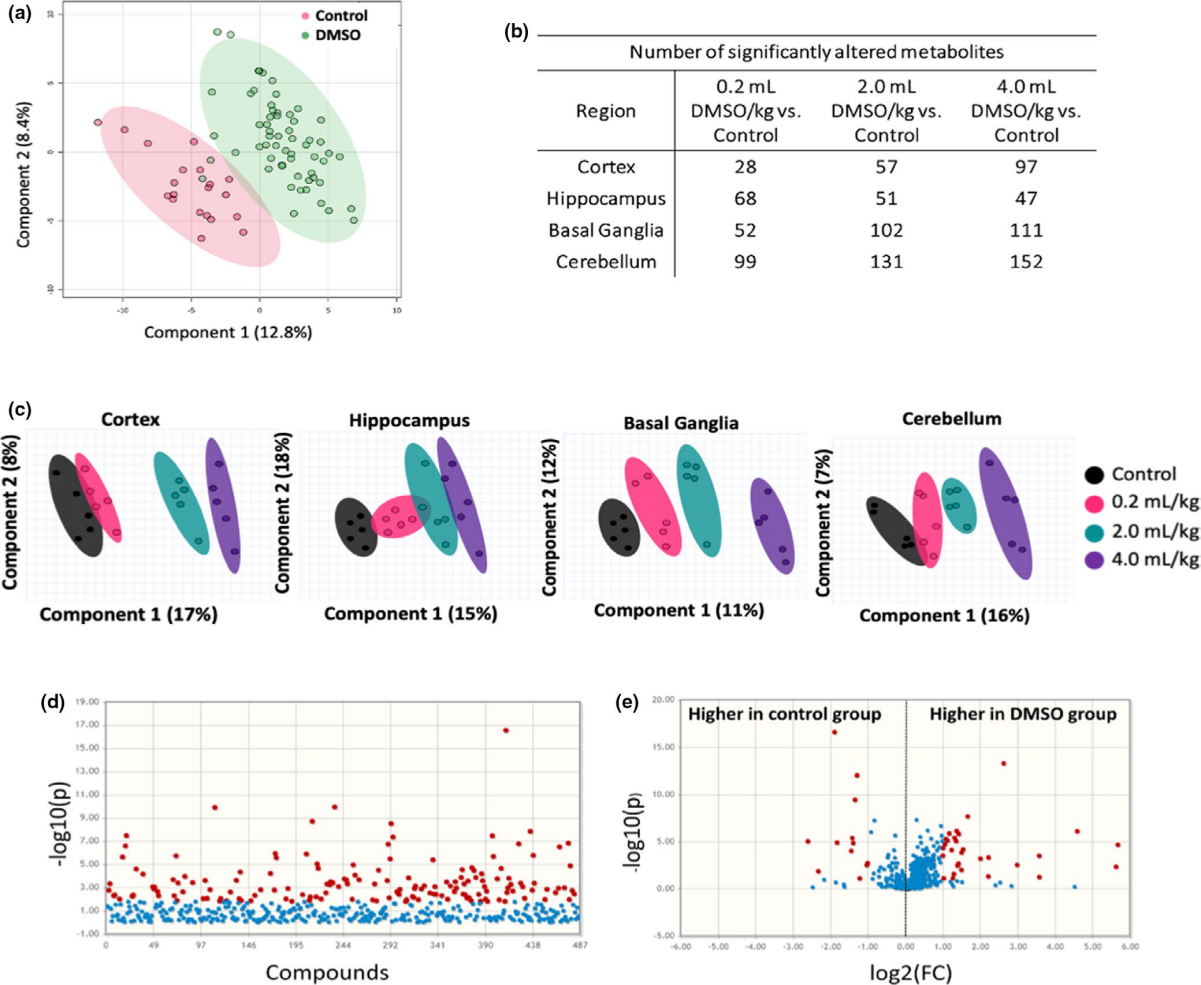
Summary of metabolites in the brain 24 hr after DMSO exposure using HILIC analysis (*n* = 20, 5 per group). (a) Principal component analysis of DMSO‐treated animals and controls. (b) Number of significantly altered metabolites by dose and region. (c) Principal component analysis of brain region and treatment. (d) Manhattan plot of metabolites of all DMSO doses and regions compared with control. Compounds in red represent metabolites with *p* < .05 (e). Volcano plot of metabolites of all DMSO doses and regions compared with control. Compounds in red represent metabolites with FDR‐adjusted *p* < .05

Principle component analysis (PCA) shows that DMSO had significant global effects on the metabolic network of the brain following any exposure to DMSO (Figure 4a). PCA shows dose‐dependent effects on the brain metabolome in each specific region (Figure 4c), and large effects were seen when analyzing only significant metabolites compared with sham‐treated control for each region and dose (Figure S3). In the 0.2 ml/kg group, the most affected brain region was the cerebellum with 99 altered compounds, followed by the hippocampus, basal ganglia, and cortex (Figure 4b). To investigate metabolites that were changed with any exposure to DMSO, regardless of region, a Wilcoxon rank‐sum test was performed (Figure 4d). 164 of the 483 metabolites were altered under DMSO treatment in at least one brain region at *p* < .05. Treatment with DMSO at any dose presented multiple significantly altered metabolites following false discovery rate (FDR) adjustment, including many compounds with more than twofold changes when compared to the sham‐treated controls (Figure 4e). Metabolic alterations varied based on dose and brain region, suggesting dose‐dependent and microenvironment effects.

Metabolites were grouped into different clusters based on their structure in order to perform chemical enrichment analysis. This clustering showed consistent changes in metabolites involved in DNA damage pathways, oxidative stress, and noncanonical metabolites. Metabolites in these clusters were further investigated to provide possible mechanistic explanations of the behavioral and cellular changes we observed.

**FIGURE 5:**
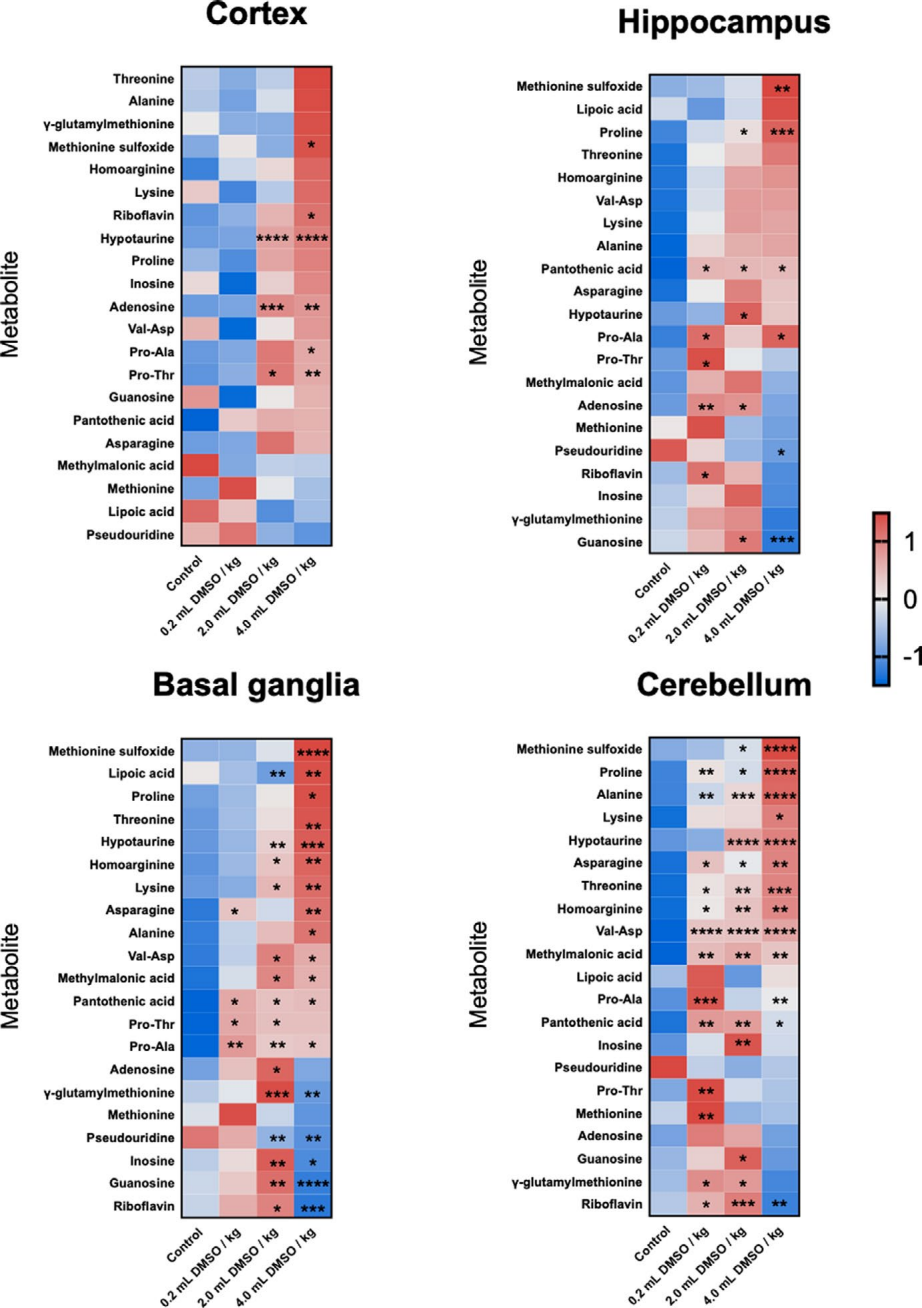
Heat map visualization of metabolites involved in nucleic acid and protein metabolism in the brain 24 hr after DMSO exposure (*n* = 20, 5 per group) measured using HILIC‐Q Exactive HF MS/MS following Z‐score transformation. *p*‐values for each metabolite are indicated by asterisks. Averages for each compound are shown to indicate fold change magnitude and direction. *p < .05 versus control (PBS); **p < .01 versus control (PBS); ***p < .001 versus control (PBS); ****p < .0001 versus control (PBS)

Nucleosides, amino acids, and oxidized metabolites showed clear dose responses and directions of regulation across the four brain regions (Figure 5). Interestingly, metabolites did not have the same response following DMSO exposure in the brain's different regions, exemplifying the spatial differences in the brain metabolome and microenvironment effects. Threonine and alanine are two examples of metabolites showing differential response by region. While both metabolites increase in a dose‐dependent fashion with DMSO, threonine is only statistically different in the cerebellum for all groups compared with the control, and alanine is only statistically altered in the highest dose of DMSO in the basal ganglia in addition to all treatment groups compared with the control group for the cerebellum. Epimetabolites such as pseudouridine are physiologically active molecules generated from adjacent canonical metabolites (Showalter et al., 2017; Zhao & He, 2015). Pseudouridine levels decreased in a dose‐dependent manner with DMSO exposure in the hippocampus, basal ganglia, and cerebellum, while levels in the cortex remained fairly constant. Guanosine had a similar trend of decreased levels in all regions of the brain following DMSO exposure. Guanosine is a neuroprotective metabolite that activates intra‐ and extracellular signaling pathways that regulate CNS functions, behavioral responses, and neuronal plasticity (Di Liberto et al., 2016). Hence, decreases in guanosine levels may have contributed to the behavioral dysfunction observed. Conversely, levels of other nucleoside and nucleotide moieties were increased in the brain following DMSO exposure (Figure 5). Protein and amino acid levels in cells are in a state of dynamic equilibrium. Increases in amino acids and dipeptides may be the result of enhanced proteasomal degradation caused by the misfolding or loss of protein structure (proteopathy) and cellular stress (Chen et al., 2017). Similarly, differences in nucleoside levels may point to dysregulation of RNA and DNA homeostasis. It was found previously that DMSO can decrease the stability of RNA and proteins by decreasing conformational structure (Tunçer et al., 2018). Verheijen et al. (2019) showed that over 2,000 microRNAs were altered when cells were exposed to as little as 1% DMSO in culture, which supports our hypothesis that DMSO induces degradation of protein and nucleic acid structures. The increase in oxidized metabolites (methionine sulfoxide and hypoxanthine) suggests that the increase in amino acids and dipeptides is due to the degradation of proteins due to loss of structure (Figure 5).

Microglia act as the resident macrophages in the central nervous system, and an increase in protein and nucleic acid catabolism would lead to cell stress, resulting in the need to clear cellular debris. An increase in catabolic debris and oxidative stress has been shown to correlate with an increase in microglia (Rojo et al., 2014). Our data showing the activation of microglia (Figure 2) support the possibility of DMSO causing oxidative stress in the brain.

**FIGURE 6:**
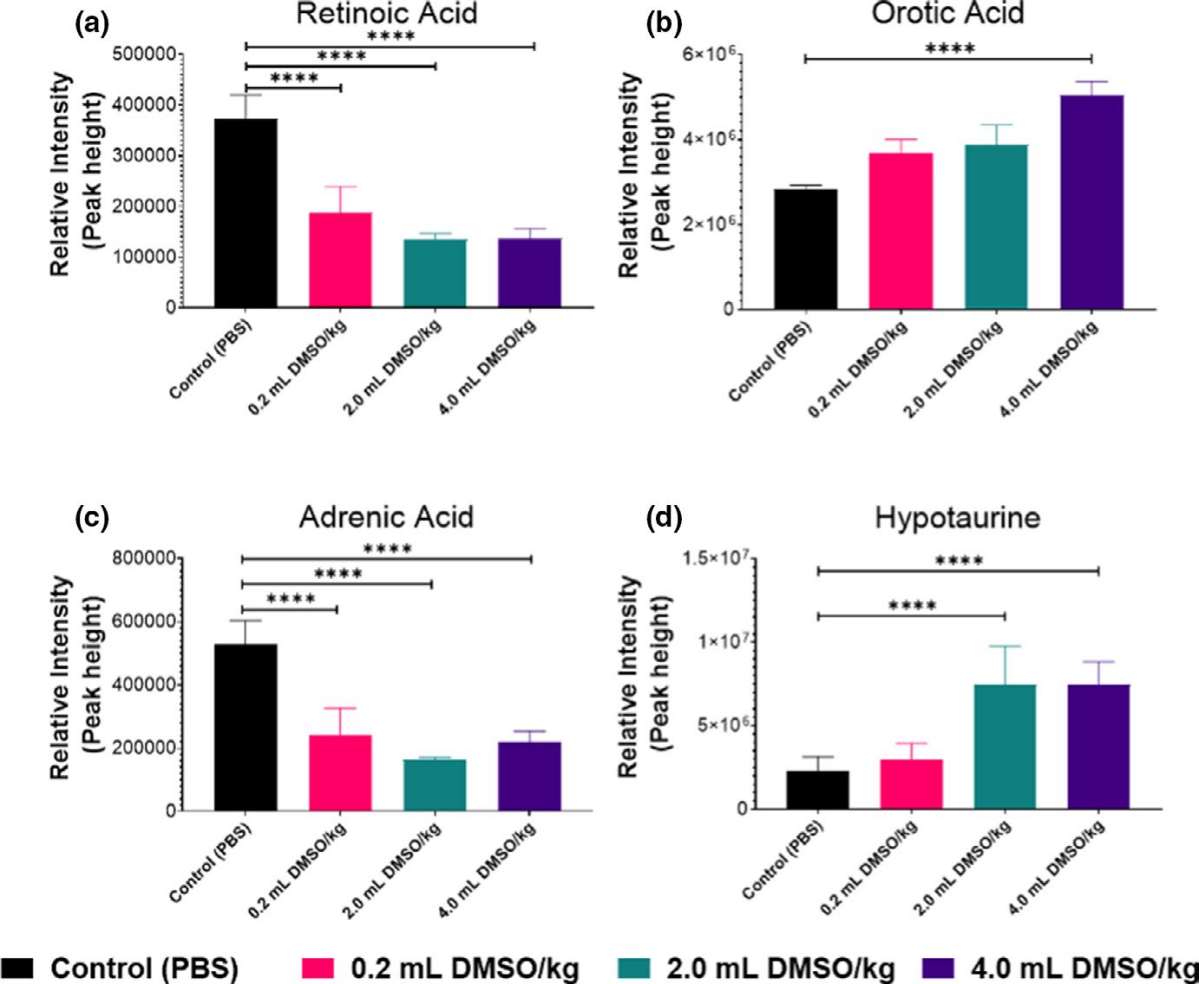
Effects of DMSO on (a) retinoic acid, (b) orotic acid, (c) adrenic acid, and (d) hypotaurine levels in whole brain following using HILIC‐Q Exactive HF MS/MS. Data are means ± *SEM* of metabolite abundance (*n* = 20, 5 per group). ****p < .0001 versus control (PBS)

Our metabolomic data showed large changes in retinoic acid (RA), the biologically active form of vitamin A, which plays a key role in gene expression, cell growth, and overall brain development. Retinoid signaling synchronizes cortical neuronal activity between separate locations in the brain, a critical phenomenon responsible for influencing sleep, memory, and learning (Dräger, 2006). RA is critical for neuronal survival and promotes antiapoptotic and pro‐proliferative activity through various pathways (Wagner et al., 2002). We have found that total RA levels have significantly decreased in a dose‐dependent manner in all DMSO treatment groups (*p* < .0001) (Figure 6a). RA levels were significantly decreased in all DMSO treatment groups in the hippocampus (*p* < .01), and basal ganglia (*p* < .001), and in the 2.0 and 4.0 ml/kg DMSO groups in the cerebellum (*p* < .03) (Figure S3a). While mechanisms have not yet been fully elucidated on how RA modulates neuronal pathways, it is hypothesized that the RA receptors or retinoid X receptors form a ligand complex and dimerize, allowing for the activation and modulation of neuronal differentiation (Jang et al., 2004). It is unclear whether the decreased levels of RA observed following DMSO exposure are due to RA acting as a ligand and being modified to retinyl esters to assist neuronal and glial cells in combating oxidative stress from DMSO, or whether RA was degraded by cytochrome p enzymes as previously described (Duester, 2008). Normal levels of RA activate the peroxisome proliferator response element of certain genes to increase protein levels of two superoxide dismutase isoforms and to preserve glial and neuronal cells from oxidative damage (Ahlemeyer et al., 2001). The marked decreased RA following exposure to DMSO in all assessed brain regions suggests DMSO causes oxidative damage, resulting in cellular stress and apoptosis, leading to the activation of microglia and astrocytes.

The nucleoside pathway precursor orotic acid (OA) was elevated in a dose‐dependent manner in all brain regions following the administration of DMSO and significantly increased in the 4.0 ml/kg treatment group (*p* < .0001) (Figure 6b). OA was significantly altered in the 4.0 ml DMSO/kg group in the cortex (*p* = .041), cerebellum (*p* = .0053), and basal ganglia (*p* = .0287) (Figure S3b). OA is produced in the mitochondria by dihydroorotate dehydrogenase, or in the cytoplasm by the pyrimidine synthesis pathway (Rawls et al., 2000). OA enables astrocytes to carry out normal aerobic metabolism even when cells were cultured under hypoxic conditions, indicating the importance of this metabolite in promoting neuronal survival during central nervous system insults or injuries (Sonnewald et al., 1998). Increased OA levels may be linked to the increase in astrocytes we observed. Furthermore, it provides an explanation on why no changes were seen in the number of neurons following brief CNS injury.

Adrenic acid (AdA) is a naturally occurring polyunsaturated fatty acid and one of the most abundant fatty acids in the developing human brain. AdA was significantly decreased in all regions following the administration of DMSO at all exposure levels (Figure 6c). AdA was significantly altered in the 4.0 ml DMSO/kg groups in the cortex (*p* = .041), cerebellum (*p* = .0053), and basal ganglia (*p* = .0287) (Figure S3c). In the brain, AdA is mainly found in myelin tissue (Martinez, 1992). AdA is formed by a 2‐carbon elongation of arachidonic acid and is further metabolized to bioactive compounds such as dihomoprostaglandins and dihomo‐EETs (Campbell et al., 1985; Yi et al., 2007). The marked decrease in AdA following brief DMSO exposure provides mechanistic insights and supports our observations in both behavioral changes and activation of microglia and astrocytes. Since DMSO exposure was carried out during the critical period of myelination in the neonatal LE rats and no changes in the number of neurons were observed, the decrease in this critical component of myelin warrants further investigation of neuronal developmental process (Duester, 2008).

Hypotaurine was consistently elevated in all regions following the administration of DMSO at 2.0 and 4.0 ml/kg doses (*p* < .0001) (Figure 6d). Hypotaurine was significantly altered in the 2.0 and 4.0 ml DMSO/kg groups in the cortex and cerebellum (*p* < .0001), in the 2.0 ml DMSO/kg group in the hippocampus (*p* = .0055), and in the 4.0 ml DMSO/kg group in the basal ganglia (*p* = .0047) (Figure S3d). Hypotaurine is an antioxidant that has been shown to increase the proliferation of cells and increase mitochondrial activity (Ha et al., 2016). Interestingly, DMSO leads to the collapse of the mitochondrial membrane potential, decreasing electron transport chain efficacy. The increased levels of hypotaurine may counteract the oxidative effects of DMSO in order to attempt to maintain normal cellular functions (Liu et al., 2001).

The alterations of metabolites discussed above suggest dysregulation of protein and nucleic acid metabolism in the brain following DMSO exposure in infancy. Alterations to RA and OA further support the notion of oxidative damage following brief DMSO exposure, which may be linked to glial cell signaling and activation. Alterations of AdA provide some insight into possible mechanism of damage to myelin, leading to the changes in behavior observed later in life after brief DMSO exposure in infancy.

## 3 CONCLUSION

**FIGURE 7:**
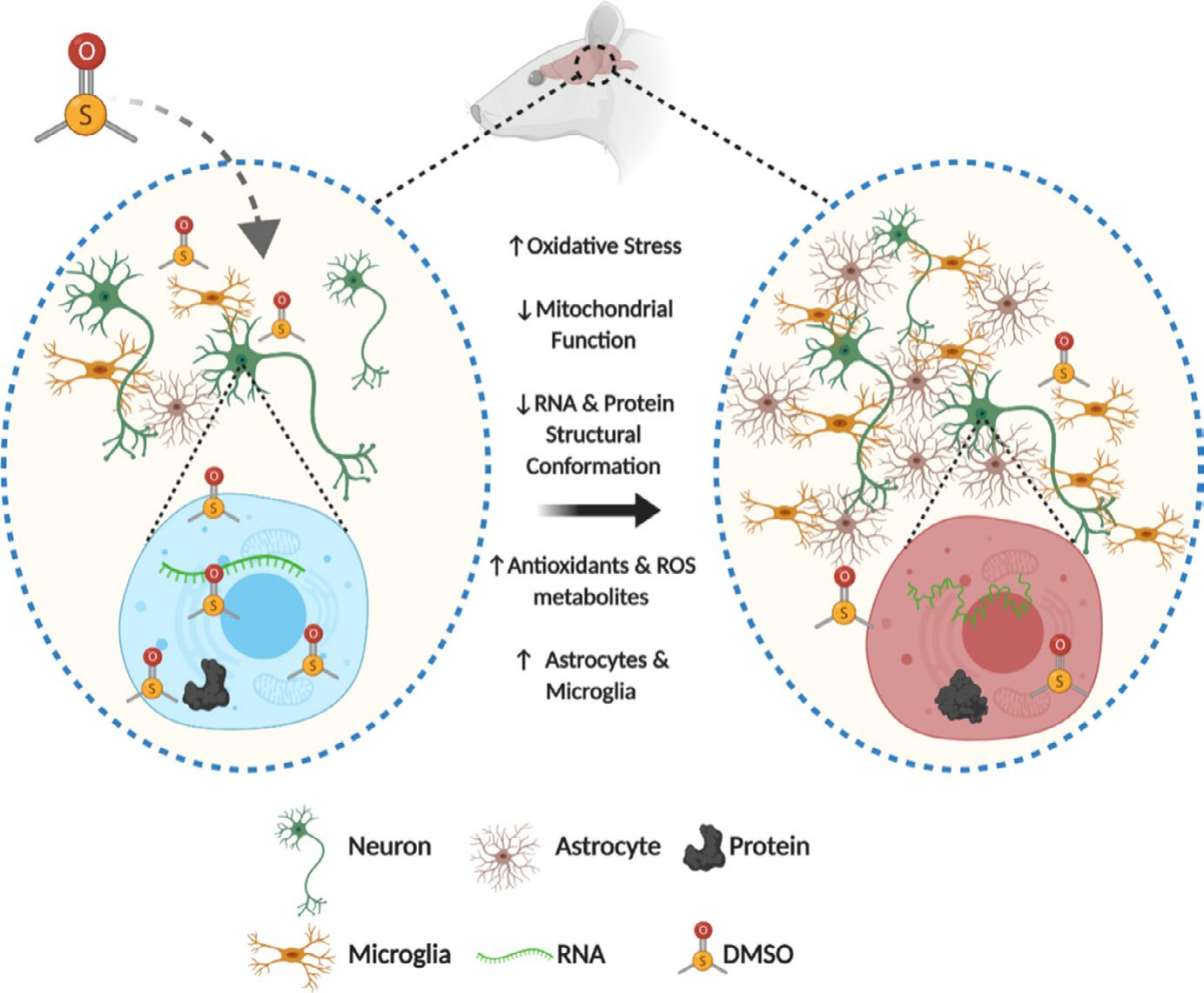
Summary of cellular and metabolic brain changes following brief exposure to DMSO

DMSO is a by‐product of algal metabolism and has been documented to be present in very low levels of fruit, but is mainly an additive in topical lotions and a cryopreservative for various medical applications (Pearson et al., 1981). Here, we unequivocally show that the administration of DMSO at previously regarded safe concentrations causes significant changes to brain biochemistry and cellular and behavioral patterns in LE rats. We observed that exposure to DMSO by IP injection at any of the low doses used resulted in global changes to the brain metabolome and an increase in both oxidative stress markers (taurine, hypotaurine, methionine sulfoxide) and proteolysis markers (dipeptides). The nonlinear relationship between DMSO administered and DMSO concentration present in the brain suggests that the glymphatic system is able to clear low levels of DMSO, but reaches a saturation point and is then unable to clear the compound. Additionally, behavioral tests in the 2.0 and 4.0 ml DMSO/kg groups have revealed hypoactive exploratory behavior and decreased social habits. Finally, we have observed a significant increase in microglia and astrocytes in brains of young adult rats following brief exposure as neonates, suggestive of chronic CNS damage (Figure 7).

We therefore propose that DMSO might be of concern in clinical practice and studies. For example, a noninterventional study carried out in 64 European centers for transplants of myeloma and leukemia showed that 95% of transplanted cells did not have DMSO removed prior to transfusion. Patients received an average of 22.6 ml over the course of two days, which equated to 0.3 ml DMSO/kg of body weight (Morris et al., 2014). Moreover, DMSO is the most common vehicle for drug delivery in high‐throughput screening models (Kenny et al., 2015). Future studies should focus on what effects sex might have on DMSO exposure and how much DMSO is absorbed from the diet in research animals. Investigation of resting and active microglia could be expanded to understand the role of glial cells following exposure; all points beyond the scope of this manuscript. Our novel findings reported here challenge the broadly perceived relative safety of low concentrations of DMSO, while observed neurotoxicity should be considered in future human medical applications and clinical trials to prevent possible chronic morphological and neurocognitive sequelae of its use.

## 4 MATERIALS AND METHODS

### 4.1 Animal experiments

Adult female Long‐Evans rats and their male pups (P4) were obtained from Charles River Laboratories. Male rats were used in order to compare our results with previously published studies that did look at the effect of DMSO exposure in female animals (Fontoura‐Andrade et al., 2017). Litters were acclimatized for 48 hr before experiments began. Treatments were distributed between preweaned litters to control for potential litter variables. Litters (i.e., dam with up to 10 pups) were individually caged under controlled temperature (22 ± 2°C) with 12‐hr light cycles (light 6 a.m.–6 p.m.; dark 6 p.m.–6 a.m.). Rat pups received IP injection with either PBS (Sigma‐Aldrich, St. Louis, MO) or DMSO (0.2, 2.0, or 4.0 ml/kg; ≥99.7% Hybri‐Max DMSO, Sigma‐Aldrich, St. Louis, MO). DMSO doses were chosen following an extensive literature search for common doses, and while not much was reported for animal experiments or in vivo studies, a 2014 report from the European Group for Blood and Marrow Transplantation documented the average dose humans received was 0.303 ml/kg body weight (±0.277 ml/kg) (Morris et al., 2014), and Long‐Evans rat pups received IP injection twice a day from P6 to P10. One cohort (*n* = 5 per group, a total of 20 animals) was sacrificed at P11. Brains were macrodissected into regions—cortex, basal ganglia, hippocampus, and cerebellum—and then snap‐frozen in liquid nitrogen and stored at −80°C until extracted for metabolomic analysis. All animals were sacrificed on the same day, and tissue was collected for each animal after cervical decapitation. The entire process took one hour, between 11 a.m. and noon, in order to minimize any diurnal fluctuation of metabolites in the brain. Animals were randomly selected, blocked by treatment group, for the order they were sacrificed. A second cohort was weaned off their dams at P20. Locomotion and behavioral assays were performed in a blinded fashion as previously described at P21 and P32 (Lechpammer et al., 2017). Animals were sacrificed at P40, and brains were preserved for morphological and histochemical analysis. All animal procedures were performed in accordance with the standards approved by the UC Davis Institutional Animal Care and Use Committee (IACUC #17420).

### 4.2 Open‐field locomotion test

The locomotor behavioral test measures animal exploration in an open‐field enclosure to assess the animal's level of anxiety or changes to their behavior (Schmitt & Hiemke, 1998). Prior to the test, each animal was introduced in the center of a 100 × 100 cm square enclosed by Plexiglas and allowed to habituate in the environment for 30 min. A day after habituation, each animal was individually placed into the same apparatus and monitored for 30 min using an overhead camera and the ANY‐maze Video Tracking System (ANY‐maze; Stoelting, Wood Dale, IL) (Crawley, 2004). The software recorded the number of times the animal entered the center zone, the animal's mean speed, the time the animal spent in the center, and the time the animal spent in the perimeter. In between trials, the chamber was cleaned with 70% ethanol.

### 4.3 Three‐chamber social choice test

Rat social behaviors were observed using “Social Approach” and “Social Novelty” tests adapted from Moy et al. (2004) as described previously by Lechpammer et al. (2017) (Lechpammer et al., 2017; Moy et al., 2004). The test apparatus was composed of two circular cages placed at the ends of a three‐chambered, Plexiglas enclosure (100 × 100 cm, with chambers 100 × 33 cm). The cages, 13.3 cm in diameter and 21 cm in height, had metal rods spaced 0.5 cm apart to allow interaction between the animal and a caged “stranger” animal. Rectangular openings between the chambers allowed the animal to move freely around the enclosure during tests.

For the social approach test, each animal was first habituated in the empty three‐chambered enclosure for five minutes. After habituation, the animal was removed, and cages were placed in the end chambers, one containing a same‐sex stranger rat, and the other left empty. The test animal was reintroduced in the center chamber and allowed to explore the enclosure for 10 min. An overhead camera and ANY‐maze software tracked time spent in each chamber. Time spent interacting with the stranger rat and the empty cage was also recorded. The expected behavior of a control rat would be to spend more time in the chamber with the stranger.

For the recognition of social novelty test, the test animal was removed from the apparatus and a novel same‐sex stranger was placed in the previously empty cage. The test animal was reintroduced to the center chamber and allowed to explore the enclosure for an additional 10 min. The same data were recorded for the test through the ANY‐maze software. The expected behavior of a control rat would be to spend more time in the chamber with the novel stranger. In between trials, the chamber and caged cylinders were cleaned with 70% ethanol.

### 4.4 Brain morphology

Serial 20‐µm coronal sections were cut by cryostat from the anterior extent of the lateral ventricles through the posterior extent of the dorsal hippocampus. Coronal sections at the level of the mid‐dorsal hippocampus were examined. Representative sections of the parietal cortex and white matter were stained by hematoxylin and eosin (H&E) or immunohistochemical and immunofluorescent labeling, as previously published (Kim et al., 2015; Lechpammer et al., 2008, 2016). Microphotographs were taken at 200× magnification, and two blinded neuroscientists counted of number of immunolabeled cells.

### 4.5 Metabolomic sample preparation

Metabolites were extracted from macrodissected fresh brain tissue (*n* = 5 for each group) as described previously (Barupal et al., 2019). Briefly, 6 mg of tissue was homogenized using 3.2‐mm‐diameter stainless steel beads ground using a GenoGrinder for 50 s at 1,500 rpm. Ground tissues were then extracted using 225 µl cold methanol containing a mixture of deuterated internal standard water and methyl *tert*‐butyl ether, as adapted from Matyash (Matyash et al., 2008). Ten method blanks were extracted and analyzed at the same time as the samples. The polar fraction of methanol and water was dried under vacuum and reconstituted in 110 µl of 80:20 (*v/v*) acetonitrile: water containing 34 deuterated internal standards. Samples were then vortexed, sonicated, and centrifuged prior to analysis.

### 4.6 LC‐MS data acquisition

Hydrophilic interaction liquid chromatography (HILIC) was used as previously described (Showalter et al., 2018). Briefly, HILIC analysis was performed using a Vanquish UHPLC coupled to a Q‐Exactive HF orbital ion trap mass spectrometer (Thermo Fisher Scientific, San Jose, CA). Chromatographic separation was achieved using a Waters BEH Amide column under the following chromatographic conditions: Mobile phase A consisted of 100% water with 10 mM ammonium formate and 0.1% formic acid. Mobile phase B was 80:20 acetonitrile: water with 10 mM ammonium formate and 0.1% formic acid. Gradients were run from 0 to 2 min at 100%B; 2–7.70 min to 70%B; 7.70–9.5 to 40%B; 9.5–10.25 min to 30%B; and 10.25–12.75 min of increase back to 100%B with column equilibration from 12.75 to 16.75 min at 100%B. The flow rate was 0.400 ml/min. The column was heated to 40°C. 5 μL of the sample was injected onto the column for analysis in both polarity modes. Data were collected from 120 to 1,200 m/z in a data‐dependent manner with the top four ions from each MS1 scan being selected for MS/MS fragmentation. Samples were randomized prior to injection with method blanks, and QC samples were analyzed between every ten study samples.

### 4.7 Data processing and statistics

Data processing was performed using MS‐DIAL v3.90 (Tsugawa et al., 2015). Raw data are available on the Metabolomics Workbench. Compounds were identified by matching retention times and experimental spectra downloaded from the HILIC‐MS/MS database in MassBank of North America in addition to NIST17 MS/MS spectra. For statistical analysis, samples were normalized to the sum of all identified metabolites. Heat maps were generated using GraphPad Prism v8.4.0. Heat map values are Z‐scores of each metabolite. Z‐scores were calculated by subtracting the mean of the group values and then dividing by the standard deviation in order to scale the data for relative expression for each region. Line graphs and violin plots were generated using GraphPad Prism v8.4.0. All *p*‐values listed for metabolites are normalized raw *p*‐values. PCA, Wilcoxon rank‐sum test, and volcano plots were generated used MetaboAnalyst v4.0. False discovery rate (FDR) adjustment was used to correct for multiple comparisons in the volcano plot. For animal experiments and immunohistochemistry data, statistical analysis was carried out using GraphPad Prism v8.4.0. All values are expressed as mean ± *SEM*. Groups were compared using the one‐way ANOVA test with the multiple comparison post hoc tests. *p* ≤ .05 were considered statistically significant.

## CONFLICT OF INTEREST

The authors declare no conflict of interests.

## AUTHOR CONTRIBUTIONS

**Zach** **Rabow** conceptualized the data, involved in formal analysis, investigated the data, curated the data, wrote original draft, and visualized the data**; Taryn Morningstar** involved in formal analysis, investigated the data, involved in behavioral studies, curated the data**,** and visualized the data; **Megan Showalter** involved in formal analysis and curated the data**; Hailey Heil** curated the data; **Krista Thongphanh** involved in formal analysis, investigated the data, and involved in behavioral studies**; Joanne Chan** investigated the data**; Sili Fan** involved in formal analysis and visualized the data; **Verónica Martínez‐Cerdeño** provided software, involved in behavioral studies, and provided resources**; David Zagzag** conceptualized the data, designed methodology, and wrote and edited the manuscript. **Robert Berman** involved in behavioral studies and provided resources**; Evgeny Nudler** conceptualized the data, designed methodology, provided resources, and acquired funding**; Oliver Fiehn** conceptualized the data, designed methodology, provided software, provided resources, wrote, reviewed, and edited the manuscript, supervised the data, administered the project, and acquired funding**; Mirna Lechpammer** conceptualized the data, designed methodology, provided software, involved in behavioral studies, provided resources, wrote, reviewed, and edited the manuscript, supervised the data, administered the project, acquired funding.

## ETHICAL APPROVAL

The vertebrate animal protocol used in this study was approved by an Institutional Animal Care and Use Committee (IACUC) at the University of California, Davis (Protocol Number: 17420).

## Supporting information

Supplementary Material

## Data Availability

The data that support the findings of this study are available from the corresponding author upon reasonable request.
