# Supplementary material for: Exposure to DMSO during infancy alters neurochemistry, social interactions, and brain morphology in long‐evans rats
Source: Brain Behav. 2021 Apr 10;11(5):e02146. doi: 10.1002/brb3.2146 (PMC8119844; doi:10.1002/brb3.2146)
Supplement: Supplementary file 1 — Supplementary Material [file BRB3-11-e02146-s001.docx]

**Exposure to DMSO in infancy alters neurochemistry, social interactions, and brain morphology in Long-Evans Rats**

Supplemental figures

**Figure S1** – Locomotion assessment of LE Rats at P21 (n= 28). Experimental groups were prepared and submitted to behavioral assessments as described in the methods. No changes were observed.


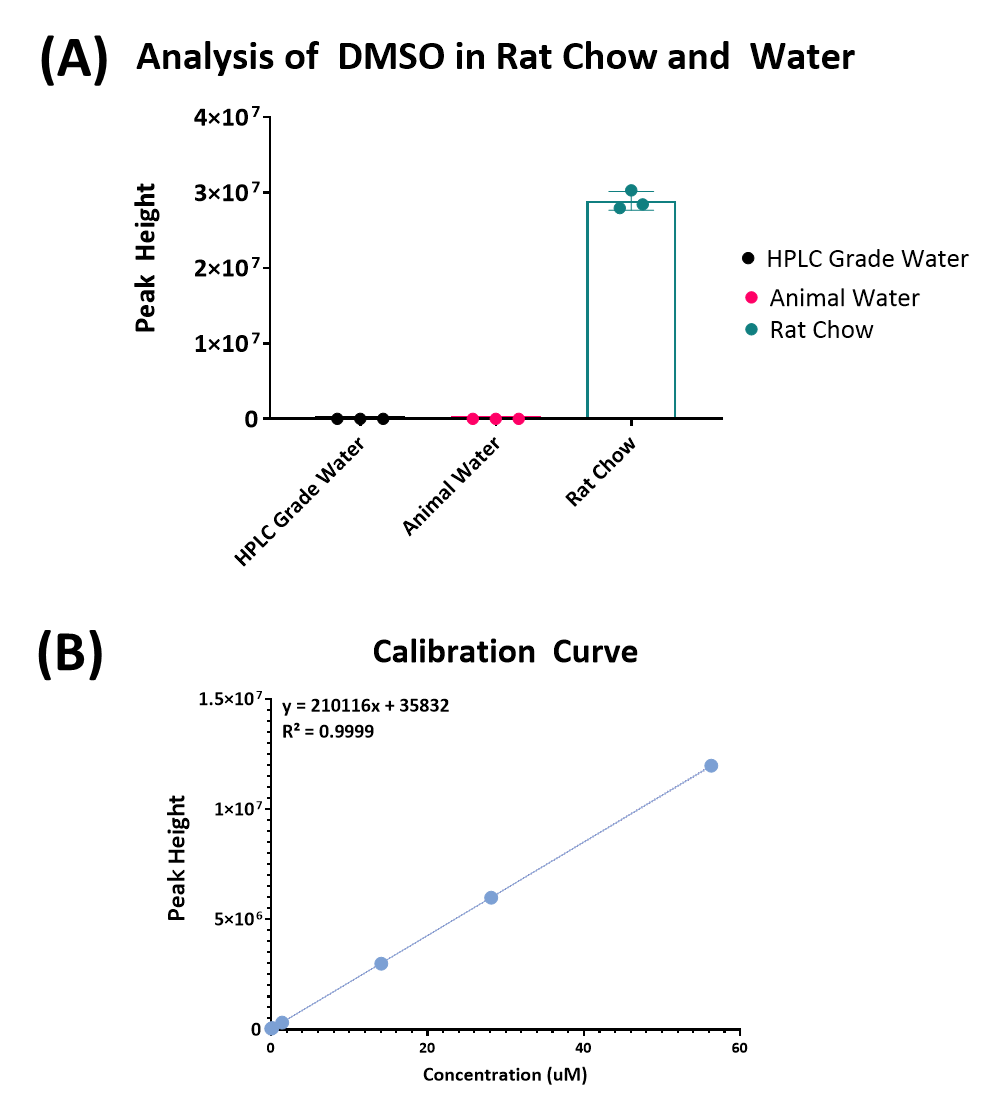


**Figure S*2-*** *Analytical precision of DMSO Detection and quantication (A) Analysis an analytical blank (HPLC grade water), animal water and food (n=3/group). (B) DMSO Calibration Curve showing excellent linearity with limit of quantificaion above 0.01 uM.*

**Figure S3** PCA plots of significant metabolites compared to sham-treated control (n= 20 total, 5 per group). A) Cortex 0.2 mL DMSO/kg vs sham-treated control B) Cortex 2.0 mL DMSO/kg vs sham-treated control C) Cortex 4.0 mL DMSO/kg vs sham-treated control D) Hippocampus 0.2 mL DMSO/kg vs sham-treated control E) Hippocampus 2.0 mL DMSO/kg vs sham-treated control F) Hippocampus 4.0 mL DMSO/kg vs sham-treated control G) Basal ganglia 0.2 mL DMSO/kg vs sham-treated control H) Basal ganglia 2.0 mL DMSO/kg vs sham-treated control I) Basal ganglia 4.0 mL DMSO/kg vs sham-treated control J) Cerebellum 0.2 mL DMSO/kg vs sham-treated control K) Cerebellum 2.0 mL DMSO/kg vs sham-treated control L) Cerebellum 4.0 mL DMSO/kg vs sham-treated control

**Figure S3 Violin plots of selected metabolites** (n= 20 total, 5 per group)*p <0.05 versus control (PBS); **p<0.01 versus control (PBS); ***p<0.001 versus control (PBS); ****p <0.0001 versus control (PBS)
